# Raynaud’s Phenomenon: A Brief Review of the Underlying Mechanisms

**DOI:** 10.3389/fphar.2016.00438

**Published:** 2016-11-16

**Authors:** Manal M. Fardoun, Joseph Nassif, Khodr Issa, Elias Baydoun, Ali H. Eid

**Affiliations:** ^1^Department of Biology, Faculty of Arts and Sciences, American University of BeirutBeirut, Lebanon; ^2^Department of Obstetrics and Gynecology, Faculty of Medicine, American University of BeirutBeirut, Lebanon; ^3^Department of Pharmacology and Toxicology, Faculty of Medicine, American University of BeirutBeirut, Lebanon

**Keywords:** Raynaud’s Phenomenon, peripheral vascular disease, alpha 2-adrenergic receptors, estrogen, thermoregulation, Rho kinase

## Abstract

Raynaud’s phenomenon (RP) is characterized by exaggerated cold-induced vasoconstriction. This augmented vasoconstriction occurs by virtue of a reflex response to cooling via the sympathetic nervous system as well as by local activation of α_2C_ adrenoceptors (α_2C_-AR). In a cold-initiated, mitochondrion-mediated mechanism involving reactive oxygen species and the Rho/ROCK pathway, cytoskeletal rearrangement in vascular smooth muscle cells orchestrates the translocation of α_2C_-AR to the cell membrane, where this receptor readily interacts with its ligand. Different parameters are involved in this spatial and functional rescue of α_2C_-AR. Of notable relevance is the female hormone, 17β-estradiol, or estrogen. This is consistent with the high prevalence of RP in premenopausal women compared to age-matched males. In addition to dissecting the role of these various players, the contribution of pollution as well as genetic background to the onset and prevalence of RP are also discussed. Different therapeutic approaches employed as treatment modalities for this disease are also highlighted and analyzed. The lack of an appropriate animal model for RP mandates that more efforts be undertaken in order to better understand and eventually treat this disease. Although several lines of treatment are utilized, it is important to note that precaution is often effective in reducing severity or frequency of RP attacks.

## Introduction

Cold-induced vasoconstriction of cutaneous arterioles is a normal physiological process that redirects blood from the superficial circulation to internal organs in order to protect the body from excessive heat loss (Charkoudian, 2010). This constriction is mediated by reflex sympathetic release of norepinephrine (Charkoudian, 2010) as well as increased sensitization of the vasculature (Vanhoutte, 1980; Wigley and Flavahan, 2016). When this cold-induced constriction is exaggerated, it leads to a pathological condition known as Raynaud’s phenomenon (RP) (Herrick, 2012). This disease can be clinically classified as primary or secondary (Block and Sequeira, 2001). Primary RP is idiopathic, and it is the most common form of the disease (Roustit et al., 2014). On the other hand, Secondary RP could be due to myriad of underlying health conditions such as autoimmune diseases or cancer, as well as lifestyle conditions such as smoking or certain medications (Prete et al., 2014). Indeed, 95% of patients suffering from Scleroderma are diagnosed with RP (Black, 1995).

Raynaud’s phenomenon affects up to 10% of the general population (Garner et al., 2015). Affected individuals suffer from cold-provoked vasospastic attacks (Heidrich, 2010) which are associated with the classic triple-color change (pallor, cyanosis, and erythema) (Maverakis et al., 2014), in addition to puffiness and ulcerations mainly at the level of fingers (Gerbracht et al., 1985). Other distal body organs such as the nose, toes, and nipples are reported to be affected (Block and Sequeira, 2001; Anderson et al., 2004). While there are different manifestations that can be used to diagnose RP, changes in some parameters may also be helpful. For example, serological tests of RP patients show increased levels of endothelin-1 (Zamora et al., 1990), tumor necrosis factor-α (TNF-α) (Rychlik-Golema et al., 2006), fibrinogen (Spengler et al., 2004), platelet factor (PF-4), and von Willebrand’s factor (vWF) (Rychlik-Golema et al., 2006). Magnesium ions and *S*-nitrosothiols levels appear to decrease in RP patients compared to unaffected individuals (Leppert et al., 1990; Kundu et al., 2014). Furthermore, anti-centromere and anti-centriole antibodies are detected in patients’ sera (Gentric et al., 1990; Yamada et al., 2014).

**FIGURE 1:**
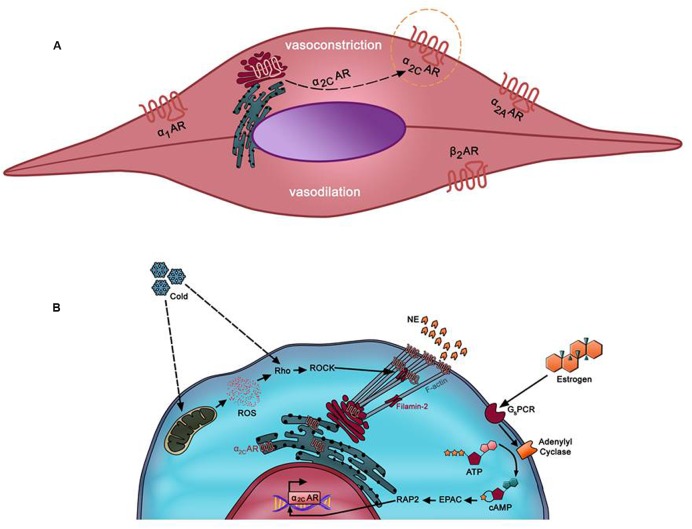
**(A)** Predominant adrenergic receptors in arteriolar vascular smooth muscle cells (VSMC). β2AR mediates mediates vasodilation of small microvessels. Vasoconstriction of these vessels occurs via α1-AR, α_2A_-AR, and α_2C_-AR. Whereas α1-, α_2A_-, and β_2_-ARs in these cells are localized at the cell surface, α_2C_-AR (in dotted orange circle) is uniquely trapped intracellularly (mostly *trans*-Golgi). However, it can be mobilized to the membrane by various stimuli such as cold temperatures. α_2C_-AR mediates cold-induced vasoconstriction, which when exacerbated may lead to Raynaud’s phenomenon (RP). **(B)** Mechanism of cold-induced mobilization of α_2C_-AR. In cutaneous arteriolar SMCs, a decrease in temperature is sensed by the mitochondria, which then releases reactive oxygen species (ROS). ROS, in turn, activates the Rho/ROCK pathway. Subsequent cytoskeletal rearrangements involving F-actin and filamin-2 promote mobilization of α_2C_-AR from the endoplasmic reticulum/Golgi to the cell surface.

Many hypotheses have been proposed to dissect and explain the underlying mechanisms implicated in the pathogenesis of RP. Recent evidence appears to lend strong support for the mosaic theory of this disease (Greenstein et al., 1996). This theory consolidates the multi-etiology of the disease, involving local, neuronal, and hormonal mediators (Wigley, 2002). Impaired function of any of these mediators may contribute to an exaggerated constriction of cutaneous arteries in response to noradrenaline (Easter and Marshall, 2005). Noradrenaline elicits its effects through binding to adrenergic receptors located on the surface of vascular smooth muscle cells (VSMCs) (Guimaraes and Moura, 2001). Typically, VSMCs have three types of adrenergic receptors (ARs): α_1_, α_2_, and β_2_. Depending on the vascular bed, β_1_ and β_3_ adrenoceptors may also be present but usually with a lower expression than β2 adrenoceptors (Ahles and Engelhardt, 2014). β_2_ adrenoceptors are involved solely in vasodilation (McCance and Huether, 2013), whereas α_1_ and α_2_-ARs are responsible for vasoconstriction (**Figure 1A**). While α_1_-ARs have a wide expression pattern across the vascular tree, α_2_-ARs are predominantly present in smaller blood vessels or arterioles (Polonia et al., 1985). At one point, these receptors were surprisingly found to be present in the protein extract of minced aortas (Chotani et al., 2004). However, histochemical analysis showed that these receptors were expressed in the *vasa vasorum* of the aorta (Chotani et al., 2004).

Early evidence clearly pointed to the prominent role of α_2_-ARs in local cooling-induced constriction of cutaneous arteries. It is important to note that local cooling causes vasodilation (Johnson and Kellogg, 2010) as well as inhibits α_1_-AR-mediated vasoconstriction (Freedman et al., 1992). Paradoxically, this very cooling also causes vasoconstriction by virtue of its potential to selectively amplify α_2_-AR-mediated constrictive effects (Jeyaraj et al., 2001; Eid et al., 2008). Because they play the key role in the sympathetic constriction of cutaneous vessels, selective potentiation of α_2_-ARs allows their cold-induced constrictive effects to overcome the vasodilatory effects. Accordingly, non-selective α_2_-AR antagonists were, at one point, used to treat peripheral cold-induced vasoconstriction but were not therapeutically effective (Freedman et al., 1993).

Molecular, genetic, and pharmacologic studies show that α_2_-ARs actually comprise three subtypes: α_2A_, α_2B_, and α_2C_ (MacDonald et al., 1997). These subtypes have their corresponding genes on three different chromosomes, and they are all coupled to inhibitory hetero-trimeric G protein (MacDonald et al., 1997). The search for the particular subtype responsible for α_2_-AR-mediated cold-induced vasoconstriction remained unclear for some time. α_2A_-ARs did not seem to play any role in cold-induced constriction (Chotani et al., 2000). Some reports pointed to the potential use of α_2B_-AR antagonists as a treatment option for vasospasms in RP (MacDonald et al., 1997). Much to our surprise, we could not find strong experimental or clinical data that support a role for α_2B_-AR antagonists in the treatment of Raynaud’s Disease.

Of the α_2_-AR subtypes, α_2C_-AR was thought to be a vestigial receptor for two main reasons. The first is that α_2C_-ARs are sequestered in an intracellular compartment (von Zastrow and Kobilka, 1994), and thus their function was not easily detected by immunohistochemistry assays (MacDonald et al., 1997). The second is that neither the α_2C_-AR knockout nor the transgenic mice showed major changes; both remained viable, fertile, and almost normal (Sallinen et al., 1997). On the other hand, other evidence emerged to argue against the apparent vestigiality of α_2C_-AR. First, α_2C_-ARs exhibit highly conserved domains present in other adrenoceptors (Nyronen et al., 2001). Second, the apparently normal phenotype may be due to compensation by other α_2_-ARs, and third, α_2C_-ARs are differentially expressed in cells of different tissues (MacDonald et al., 1997).

One interesting and rather unique feature of its biology is that upon certain physiologic and pathophysiologic stimuli, α_2C_-AR can translocate from the endoplasmic reticulum (ER) and Golgi apparatus to the cell membrane. This spatial rescue renders the receptor available for its ligand, whose binding then activates the receptor (Chotani et al., 2000, 2004; Jeyaraj et al., 2001). Upon moderate physiological cooling (i.e., 28°C), α_2C_-AR is mobilized from the ER/Golgi to the cell surface (Bailey et al., 2004). The now membrane-localized receptors can readily interact with their agonists, become activated and evoke cutaneous vasoconstriction in response to norepinephrine (Jeyaraj et al., 2001). Indeed, it is now evident that the entirety of cold-induced constriction of cutaneous arteries is due to an increased activity of α_2C_-ARs (Bailey et al., 2004; Eid et al., 2008). As such, α_2C_-ARs appear to play an important role in the augmented vasoconstriction observed in RP (Bailey et al., 2004).

The mechanism by which α_2C_-AR translocation takes place involves different players such as reactive oxygen species (ROS), Rho/Rho kinase, and the actin cytoskeleton (**Figure 1B**). Bailey et al. reported that the Rho/Rho kinase pathway becomes activated as early as few minutes after cells get exposed to cold temperatures (Bailey et al., 2004). The now active Rho evokes the mobilization of α_2C_-AR to the membrane, and consequently triggers cold-induced vasoconstriction (Bailey et al., 2004). In this sense, it seems that Rho, rather than α_2C_-AR, is the “thermosensor” (Bailey et al., 2004). However, additional and rather elegant investigations from the Flavahan group further showed that the mitochondrion is the “thermo-sensitive” organelle in VSMCs (Bailey et al., 2005). Indeed, upon cold stress, it is the mitochondria that initiate the process by releasing ROS, which in turn triggers a redox signal that activates the Rho/Rho kinase pathway leading to spatial redistribution and functional activation of α_2C_-ARs (Bailey et al., 2005). This cooling-induced Rho activation may then act through calcium sensitization or via modulation of cytoskeletal architecture (Hall, 1998; Jeyaraj et al., 2001; Chitaley and Webb, 2002).

## RP and the Actin Cytoskeleton

The cytoskeleton plays a major role in fundamental cellular processes like cell division, migration, cell-cell communication, and protein trafficking (Fletcher and Mullins, 2010). The translocation of α_2C_-ARs, a main player in RP, from the ER/Golgi to the cell membrane of VSMCs is critical for their activation. This translocation involves many cytoskeletal components such as F-actin and actin/myosin filaments. It is through modulation of the actomyosin filaments that VSMC contraction and ultimately vasoconstriction occur.

Cold-induced, Rho-mediated architectural change occurs by virtue of a rearrangement of the actin superstructure evident by an increase in F-actin, a downstream effector of Rho kinase signaling (Jeyaraj et al., 2012). Interestingly, immunocytochemical analysis shows that intracellular α_2C_-AR and F-actin are sometimes found to be co-localized in non-vascular cells (Hurt et al., 2000). In a rather elegant and orchestrated series of events, α_2C_-ARs then get in close proximity and associate with actin filaments, readying themselves for the trafficking process (Jeyaraj et al., 2012). This intimate association appears to be mediated by a direct interaction between α_2C_-ARs and filamin-2, a cross-linker of actin filaments (Motawea et al., 2013). Indeed, further *in silico* protein-protein docking examinations confirmed that the interaction between α_2C_-AR and F-actin occurs via the direct binding of α_2C_-AR to filamin, the actin binding protein (Pawlowski et al., 2014). Interestingly, this interaction has evolved only in warm blooded animals (Pawlowski et al., 2014). Therefore, elucidation of similar protein-protein interactions can help establish more efficient therapies for exaggerated vasoconstriction. One scenario would include approaches that seek to disrupt the interaction between α_2C_-AR and the cytoskeletal component, F-actin.

## RP and Estrogen

**FIGURE 2:**
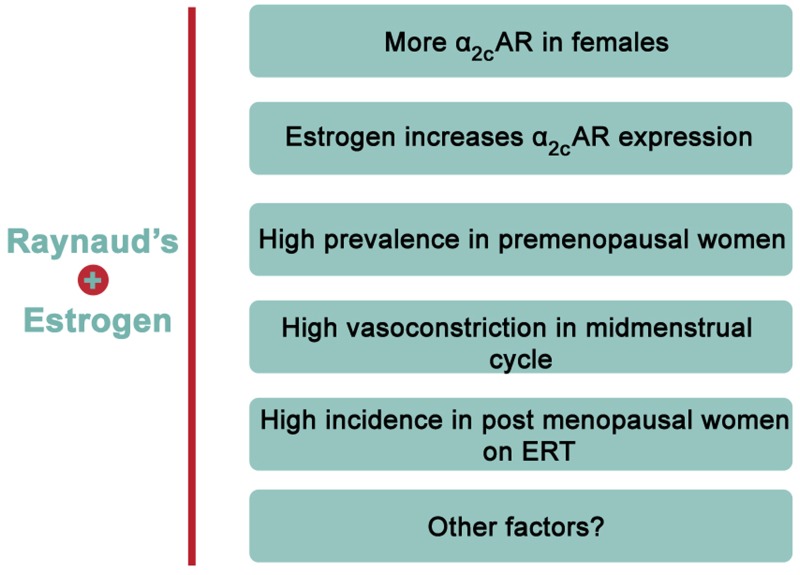
**Evidence of positive association between estrogen and RP.** Accumulating evidence points to an overwhelming association between estrogen and RP. For instance, estrogen increases α_2C_-AR but not α_2A_-AR in human arteriolar smooth muscle cells. Moreover, females have higher expression of α_2C_-AR than males. Epidemiologically, RP is reported to have remarkably high incidence in premenopausal females or post-menopausal females on estrogen replacement therapy (ERT).

Evidence from epidemiological studies reveals a rather interesting finding regarding the prevalence of RP. There is a significantly higher incidence of this disease in females versus age-matched males (Maricq et al., 1993; Garner et al., 2015). Indeed, 70% of all American patients suffering from RP are females (Maricq et al., 1993). Among patients affected with RP, the ratio of premenopausal females compared to age-matched males is close to 9:1 (Belch and Ho, 2001). This clearly illustrates a gender-based element in the prevalence of the disease, and thus hints to a potential role of sex hormones in its onset or pathology (Maricq et al., 1993). Although it is reported that cardiovascular diseases in general are more prevalent in men and post-menopausal women (Reslan and Khalil, 2012), being a female is among the risk factors of RP (Garner et al., 2015). This conclusion is partly based on a meta-analysis study asserting the much higher prevalence in females compared to males (Garner et al., 2015). In particular, the incidence is higher in premenopausal versus post-menopausal women, with an interesting association between the menstrual cycle and cold-modulated digital blood flow (Greenstein et al., 1996). Further analysis revealed that post-menopausal females receiving unopposed estrogen replacement therapy (ERT) are more likely to suffer from the disease than post-menopausal women that are not receiving ERT (Mayes, 1999). Together, these findings demonstrate that estrogen may explain the higher incidence in premenopausal women (**Figure 2**). Interestingly, in post-menopausal women receiving opposed estrogen therapy (estrogen and progesterone together), the incidence of RP was not significantly higher than that in premenopausal women (Fraenkel et al., 1998). This may suggest that progesterone negates estrogen’s effect in this context, but this remains to be established.

It is worth mentioning that in premenopausal females, noradrenaline-mediated vasoconstriction is higher at the mid-menstrual cycle, characterized by relatively high estrogen level, than during the early stage of the cycle (Chan et al., 2001). Moreover, human and rat females of reproductive age exhibit higher vascular responsiveness than males (Li et al., 2014). Interestingly, male vascular responsiveness is potentiated when 17β-estradiol is externally supplemented (Li et al., 2014). This implies that estrogen has a direct effect on vasoreactivity, though the mechanisms for such potentiation remain unclear.

The fundamental role of estrogen in regulating body temperature has been defined (Charkoudian and Stachenfeld, 2016). Although estrogen has a vasodilatory effect, it may in many instances decrease body temperature (Charkoudian and Stachenfeld, 2016). Since RP can be considered a vascular thermoregulatory control disorder (Flavahan, 2015), the implication of estrogen in the disease becomes obvious especially in light of the exaggerated response to cold in premenopausal women as well as the higher prevalence of RP in younger females. This is further supported by the findings of English et al. that there is a gender difference in vasomotor activities in response to estrogen, and that this difference may be a critical contributor to the etiology of vasospastic diseases (English et al., 2001), such as RP.

Evidence indicates that estrogen increases α_2C_-AR expression in VSMCs and that α_2C_-AR-mediates cold-induced vasoconstriction in rat tail arteries (Eid et al., 2007). A notable finding is that among the α_2_-ARs, only the α_2C_-AR subtype is differentially expressed in rat tail arteries, with a remarkably greater expression in females (McNeill et al., 1999). We had also reported that in human VSMCs, estrogen does not modulate the expression of α_2A_-AR (Eid et al., 2007). The Flavahan group had also established that α_2C_-AR mediates the entirety of cold-induced vasoconstriction. We then hypothesized and later confirmed that estrogen indeed increases the expression, surface-localization, and function of α_2C_-AR (Eid et al., 2007). This estrogen-induced activity of α_2C_-AR was followed by a potentiated cold-induced vasoconstrictive response in mouse tail arteries (Eid et al., 2007). Collectively, these pieces of evidence highlight a positive association between estrogen and RP.

## RP and Genetic Background

**FIGURE 3:**
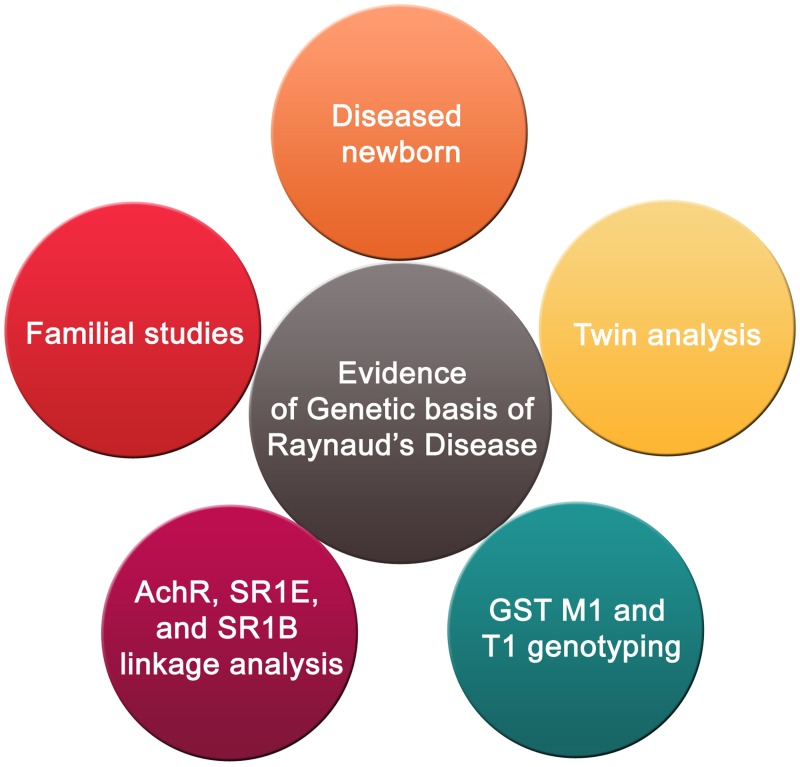
**Genetic basis of RP.** The genetic basis of RP is supported by familial studies and twin analysis in addition to a reported case of a 1-month male baby diagnosed with the disease. Furthermore, a combination of positive genotypes for both genes encoding glutathione *S*-transferase M1 and T1 subtypes may have a role in susceptibility to RP. Linkage analysis pinpointed five areas corresponding to three candidate genes (β-subunit of muscle acetylcholine receptor, 1E and 1B serotonin receptors) which could be associated to RP.

As mentioned earlier, RP is either idiopathic, or secondary to another disease like scleroderma. There have been some speculations that genetic predisposition may be a contributor to the onset of this disease (Tan and Arnett, 2000) (**Figure 3**). However, sequencing results showed no mutations in candidate genes that are suspected to play a role in the etiology of the disease (Susol et al., 2000). These candidate genes are the beta subunit of the muscle acetylcholine receptor and the serotonin 1B and 1E receptors (Susol et al., 2000). Nonetheless, others continued to suggest that there is a genetic factor contributing to the prevalence of this disease (Pistorius et al., 2006). This assertion is supported by familial studies and twin analysis (Pistorius et al., 2006). Recently, there was a reported case of a 1 month male baby diagnosed with RP (Sharathkumar and Castillo-Caro, 2011). In light of this case, it was speculated that there could be a genetic basis of the disease. However, much evidence remains lacking before a strong causative link between genetics and RP can be affirmed.

Interestingly, studies of RP patients that were exposed to vinyl chloride monomer (VCM) suggest that the interaction between a certain genetic background and environmental conditions may play a role in increasing the onset of RP in VCM-exposed individuals (Fontana et al., 2006). In 2006, Fontana et al. (2006) investigated whether there is an association between polymorphisms in glutathione *S*-transferase M1 and T1 genes and RP patients exposed to VCM (Fontana et al., 2006). The results showed that the combination of positive genotypes for both genes may increase susceptibility to RP (Fontana et al., 2006). In another study, using 298 microsatellite markers, a two-stage whole genome screen of six extended families having at least three RP patients in each family was undertaken (Susol et al., 2000). Linkage analysis identified five chromosomal areas of possible linkage. These were mapped to three candidate genes (β-subunit of muscle acetylcholine receptor, 1E and 1B serotonin receptors) which could be associated with RP (Susol et al., 2000). This provides evidence of a genetic basis for RP susceptibility. The fact that five possible linkages were highlighted indicates that RP may be an oligogenic rather than monogenic condition. However, more research is needed to ascertain this suggestion, since some of the findings reported may be false positives (Susol et al., 2000). It would, therefore, be interesting to screen in a large pool of RP patients, for mutations or SNPs in these candidate genes.

## Toxicological Basis of RP

Many of the heightened vasoreactivity responses observed in RP are due to either sympathetic or local causes. Stressors such as cold temperatures or emotional anxieties fall under the sympathetic category, since they cause vasoconstriction via noradrenaline. On the other hand, mechanical and chemical stresses fall under the “local” category since they directly affect a body organ that will show symptoms of the disease. A prominent body area that could be affected by these “local” insults would be the digits. Prolonged exposure to vibration at the level of the hand and arm is an example of mechanical stress. Also known as vibration-induced white finger, this hand-arm vibration syndrome is indeed one form of secondary RP that is due to occupational hazards (White et al., 2004). Continuous insults of the hand and arm by vibrating machines can prime these organs for increased vasospastic attacks upon a thermal or emotional stress. With the progression of this condition, such vibration can cause increased digital vasospasm even at room temperature (White et al., 2004). Therefore, it is not surprising that vascular symptoms are highly prevalent among workers whose job requires handling vibrating tools (White et al., 2004).

One of the prominent examples of chemical stressors in RP is VCM. This monomer is a colorless gas used in the manufacturing of plastic, particularly poly vinyl chloride (PVC). Interestingly, almost one third of workers exposed to PVC suffer from RP (Maricq et al., 1978). Angiography of these patients’ hands showed vascular tone changes and vascular lesions such as narrowing of the digital arteries (Falappa et al., 1982). This is not surprising since angiographic and capillaroscopic examinations have shown that exposure to VCM is toxic for the endothelium (Maricq et al., 1976; Falappa et al., 1982). Furthermore, exposure to VCM was shown to significantly contribute to acroosteolysis of distal phalanges of hands, which was recurrently associated with symptoms of RP (Wilson et al., 1967). Indeed, and as mentioned earlier, a higher prevalence of RP among French workers exposed to PVC was reported (Fontana et al., 2006). Taken together, these observations support the notion that a persistent toxic effect of polyvinyl chloride can contribute to the onset and pathogenesis of RP.

There are other chemical compounds or even medicinal drugs that are linked to the onset of RP. Some examples include arsenic, nicotine, and the drug gemcitabine. Indeed, a positive correlation seems to exist between Arsenic and RP. A study in Chile shows that increased prevalence of signs and symptoms of peripheral vascular disease, including RP, are associated with Arsenic-contaminated drinking water (Nordberg et al., 2014). Contextually, Arsenic-exposed smelter workers exhibit heightened vasospastic reactivity in the fingers, reminiscent of RP (Lagerkvist et al., 1986; Hall, 2002; William and Markowitz, 2007). Together, these findings provide some evidence of arsenic being a player in the etiology of RP.

Smoking has been long found to positively associate with RP (Garner et al., 2015). Moreover, nicotine, one main constituent in tobacco, is known to significantly decrease blood flow and increase vascular resistance (Cardelli and Kleinsmith, 1989). It is thus not surprising that nicotine can exacerbate symptoms of RP (Cherniack et al., 2000; Jackson, 2006), so much so that avoiding nicotine has been suggested as one element in the treatment of RP of the nipple (Anderson et al., 2004).

Some drugs such as gemcitabine, a nucleoside analog used in chemotherapy (Carmichael, 1998), could evoke symptoms reminiscent of RP (Yamada et al., 2014). Indeed, when orally administered, it appears to cause pain, swelling, and whitening of the digits, all of which are typical of RP (Carmichael, 1998). Indeed, a case of RP and digital necrosis after receiving gemcitabine for bladder cancer has been reported (D’Alessandro et al., 2003). Furthermore, a scleroderma patient developed digital ischemia after receiving a combined treatment with gemcitabine and carboplatin (Clowse and Wigley, 2003). Interestingly, the association of gemcitabine chemotherapy with digital ischemic events appear to be more common than previously suspected, especially in patients with tobacco-associated cancers (Kuhar et al., 2010). While the mechanisms for this gemcitabine-induced vascular insult remain unclear, it is proposed that endothelial damage as well as thrombotic microangiopathy (Venat-Bouvet et al., 2003; Holstein et al., 2010) may be contributing factors. It is important to note that this gemcitabine-associated vascular toxicity is rather pronounced in scleroderma patients. Similarly, exacerbations of RP symptoms were found to be associated with fluoropyrimidine, namely capecitabine, therapy (Coward et al., 2005). As such, caution should be taken when administering chemotherapeutic agents, especially gemcitabine, to scleroderma or RP patients.

Chemotherapeutic agents, other than gemcitabine, have also been associated with RP. For instance, doxorubicin, and cyclophosphamide-induced scleroderma cases mostly present with diffuse sclerosis and RP (Saif et al., 2016). Moreover, vincristine-induced dose-dependent RP has been reported (Gottschling et al., 2004). Interestingly, a higher prevalence of RP is noted when cisplatin is combined with vinblastine (Vogelzang et al., 1981). Whether the neurotoxic effects of these drugs underpins the increase RP prevalence remains poorly determined. It has been suggested that hyperreactivity in the sympathetic outflow may be an underlying cause (Chant, 1987; Olsen et al., 1987). However, this cannot exclude the possibility that a direct effect on the vasculature of terminal arterioles is also possible, especially that the entirety of cold-induced vasoconstriction is mediated by vascular α2C-AR, independently of any contribution from the endothelial or the sympathetic nervous system (Kristensen, 1979; Eid et al., 2007, 2008; Wigley and Flavahan, 2016).

Epidemiologic data derived from a long-term study involving combined treatment with cisplatin, vinblastine, and bleomycin chemotherapy showed that 35–45% of these treated patients developed RP (Hansen and Olsen, 1989). Bleomycin, in particular, appears to be the key player in the development of RP in these patients. Indeed, findings of a recent large cross-sectional study showed that the only significant predictor of persistent RP at follow-up after chemotherapy was the bleomycin dose (Glendenning et al., 2010).

## Treatment of RP

Significant efforts have been undertaken to better understand and treat RP (Lee et al., 2014; Poredos and Poredos, 2016). However, no definitive or specific therapy for this disease has yet been approved by the U.S. Food and Drug Administration (FDA; Landry, 2013). One of the limiting factors in the war against this disease in the incomplete understanding of its pathophysiology (Landry, 2013), which is further compounded by the lack of appropriate animal models for RP. Moreover, a treatment regimen efficacy may depend on the severity and type of the disease, as well as on the degree of vascular damage. Despite that, some medications or treatment options that are thought to alleviate symptoms of the disease are being employed in the clinic. These options can be collectively classified into traditional pharmacological, ethno-pharmacological, non-traditional treatments, and most recently surgical intervention (**Table 1**).

**Table 1:** Various lines of treatment of Raynaud’s disease.

	Treatment	Effectiveness	Reference
Traditional treatment	Calcium channel blockers	Effective; first-line of treatment	Halawa, 2001; Thompson and Pope, 2005
	PTK inhibitors	Efficient	Furspan et al., 2004, 2005
	PDE5 inhibitors	Inefficient	Lee et al., 2014
	Beta-blockers	Controversial	Marshall et al., 1976 Koltringer et al., 1991
	Statins	Emerging/powerful	Abou-Raya et al., 2008
	Prostacyclins	Efficient	Rademaker et al., 1987
	ACE inhibitors	Variable effect	Henness and Wigley, 2007
	Endothelin receptor antagonists	Variable effect	Poredos and Poredos, 2016
	Serotonin receptor antagonists	Effective	Coleiro et al., 2001
Non-Traditional treatment	Botulinum toxin type A	Efficient	Neumeister et al., 2014
	Chinese herb	Ineffective	Wu et al., 2008
	Ginkgo biloba	Ineffective	Muir et al., 2002
	Acupuncture	Efficient	Appiah et al., 1997
	Laser therapy	Efficient	Hirschl et al., 2004
Surgery	Thoracic sympathectomy	Effective	Coveliers et al., 2011
	Hand stripping	Effective	Balogh et al., 2002
	Nerve stimulation	effective	Kaada, 1982
	Fat grafting	Encouraging results	Bank et al., 2014

*A summary of the traditional and non-traditional therapies, as well as some surgical interventions*.

Traditional pharmacological drugs alleviate RP symptoms by reducing vasoconstriction, inducing vasodilatory effect, or by a yet unclear mechanism. Drugs used for a vasodilatory effect include calcium channel blockers, cyclic guanosine monophosphate (cGMP)-specific phosphodiesterase type-5 (PDE5) inhibitors, prostacyclins, prostaglandin analogs, and alpha-1 blockers. Calciumchannel blockers are the most common and preferred first-line treatment (Halawa, 2001; Thompson and Pope, 2005). However, in the most recent and highly comprehensive Cochrane review where seven randomized trials involving 296 patients were analyzed, it was concluded that oral calcium channel blockers are relatively ineffective in the treatment of primary RP. Authors of this important review conclude that evidence does not support a role of these blockers in reducing the frequency and severity of attacks (Ennis et al., 2016). This is somewhat inconsistent with an earlier meta-analysis which suggested some, albeit small, efficacy of these blockers in reducing the severity of RP attacks (Thompson and Pope, 2005). However, authors of this paper highlighted the notion that most of the trials included in their meta-analysis were crossover studies that did not determine order effect, thus likely introducing some bias (Thompson and Pope, 2005). cGMP-specific PDE5 inhibitors have been used as well (Caglayan et al., 2006). Indeed, in an open-label pilot study involving 40 patients, it was found that digital flow was significantly improved in RP patients receiving the PDE V inhibitor, vardenafil, treatment (Caglayan et al., 2006). Consistently with this, it was also found that PDE5 inhibitors decrease vasospastic attacks and improve digital blood flow (Lee et al., 2014). Indeed, this efficacy of PDE5 inhibitor was reported in a double-blind, randomized, cross-over study involving 29 patients that were divided into two groups. One group received udenafil, a PDE V inhibitor, and the other a calcium channel blocker, amlodipine, over a period of 4 weeks. Both treatments showed comparable efficacy in RP treatment in regard to decreasing the severity of vasospastic attacks (Lee et al., 2014). In addition, patients receiving the PDE V inhibitor showed better digital blood flow when compared to those receiving amlodipine (Lee et al., 2014).

Prostanoids are reported to decrease the severity and frequency of vasospastic attacks in RP patients. Their efficacy has been consistently reported in systematic reviews, meta-analyses as well as in multiple randomized clinical trials (Clifford et al., 1980; Mohrland et al., 1985; Wigley et al., 1994; Pope et al., 2000; Scorza et al., 2001; Milio et al., 2006; Kawald et al., 2008). For instance, iloprost, a prostacyclin analog was used to treat 13 patients with RP. In addition to reducing ulcerating lesions, iloprost also caused improvement in blood flow in these patients (Rademaker et al., 1987). Moreover, in a double-blind placebo-controlled trial, it was found that buflomedil causes a reduction in the frequency of attacks, but with no effect on Raynaud severity score (Le Quentrec and Lefebvre, 1991). On the other hand, a Cochrane review concluded that evidence does not support a benefit for beraprost, ketanserin, dazoxiben, and moxisylyte in ameliorating frequency duration or severity of attacks (Stewart and Morling, 2012). However, the authors of this review indicated that the precision of their conclusion is affected by the fact that most of the studies included in their review are poorly designed or executed

Angiotensin receptor blockers, ACE (Angiotensin Converting Enzyme) inhibitors, PTK (protein tyrosine kinase) inhibitors, and endothelin receptor antagonists (ETRAs) are also employed in the treatment of RP owing to their ability to reduce vasoconstriction. A clinical trial reported that losartan (50 mg) causes a significant reduction in the severity and frequency of spastic episodes (Dziadzio et al., 1999). The therapeutic benefit of using ACE inhibitors in the management of RP seems to be variable (Henness and Wigley, 2007). Some studies have reported that they may have minor benefits, albeit to a lesser extent than traditional therapies (Wood and Ernst, 2006). Indeed, it is not recommended that angiotensin receptor blockers be replaces with ACE inhibitors for the treatment of RP (Linnemann and Erbe, 2016). It is important to note here that although enalapril and captopril are reported to reduce the number of attacks in primary RP, they do not appear to be effective in reducing these attacks in secondary RP (Tosi et al., 1987; Janini et al., 1988). Moreover, in a multicenter, randomized, double-blind, placebo-controlled trial involving 210 patients, quinapril treatment for up to 3 years did not show any beneficial effects in reducing the severity of frequency of attacks (Gliddon et al., 2007). The increased phosphorylation of PTK is associated with the α_2C_-AR-mediated vasoconstriction, thus PTK inhibitors may be used to reverse the contractile response to cooling, as these studies show that PTK phosphorylation is higher in RP patients arterioles in comparison to ctrl arterioles (Furspan et al., 2004, 2005). However, future studies must be done on this interesting type of treatment.

One of the early events thought to play a role in the vasculopathy of scleroderma is endothelial injury. Because such injury leads to increased release of the potent vasoconstrictor, endothlin-1, it was thought that blocking endothelin signaling and function could play a beneficial role in the treatment of systemic scleroderma (SSc) and the associated secondary RP. When ETRAs were employed, not all patients responded positively; nonetheless, these antagonists were able to at least alleviate the severity and frequency of vasospastic attacks (Poredos and Poredos, 2016). Several studies have looked at the effect of ETRAs in the treatment of SSc-associated RP. In 2006, the first prospective study investigating the potential benefit of ETRAs in RP was published (Selenko-Gebauer et al., 2006). The patients involved in this study received bosentan for 16-week, after which it was found that severity of RP attacks was significantly reduced. Another observational study also reported that after a median of 8 weeks of treatment with bosentan, severity of RP was also reduced (Funauchi et al., 2009). Whether beta blockers have a therapeutic value remains controversial. One studies involving 102 patients report that the beta blockers Propranolol, Oxprenolol, and Atenolol disturb the microcirculation causing RP as a side effect (Marshall et al., 1976). Consistent with this, a meta-analysis of 13 studies suggests that the use of beta blockers is associated with higher incidence of RP (Mohokum et al., 2012). On the other hand, other reports suggest that beta blockers could be beneficial particularly because of their ability to reduce blood viscosity (Koltringer et al., 1991). In this study, half of the 40 participants involved received metoprolol, and showed reduced blood viscosity compared to the control group. Interestingly, a combination treatment of beta blockers (metoprolol) with calcium channel inhibitors (felodipin) was shown to be very effective in reducing symptoms of RP (Csiki et al., 2011).

There are other drugs that appear to have a potential for use in the management of RP. These include statins (Abou-Raya et al., 2008) and serotonin receptor antagonists (Coleiro et al., 2001). Although their mechanism of action is not fully clear, they appear to retard vascular injury, lessen severity, and reduce pain associated with RP.

A recent report discussed the potential benefit of using a rather non-traditional approach for the treatment of RP. Botulinum toxin type A (BTX-A) can be locally injected to improve ulcerated digits and alleviate the associated pain (Neumeister et al., 2014). This improvement may be due to better perfusion and improved vascularity; however, the exact mechanism remains unknown. Notably, studies have shown that the use of BTX-A could be safe and efficient (Neumeister et al., 2014). In a recent retrospective study, it was shown that local injection of BTX-A provides great improvement in artery flow velocity, surface temperature, ulcer, and other clinical symptoms (as measured by visual analog scale; Zhang et al., 2015). Others have also reported similar beneficial effects of BTX-A in the management of RP (Smith et al., 2012; Zhao and Lian, 2015). However, despite all these interesting and promising results, a recent systemic review concludes that evidence to support the efficacy of BTX-A in the management of RP remains insufficient. As such, further research, particularly randomized controlled trials, is needed to better determine the potential efficacy of this interesting approach.

It is worth mentioning that in some patients, the aforementioned pharmacological drugs may cause several side effects such as headaches and dizziness. As such, many patients resort to alternative therapies in the hope of avoiding such undesired side effects. Herbal therapies are one common approach. Of particular interest in the management of RP is Ginkgo biloba plant extracts (Muir et al., 2002) or a combination of two Chinese herbal medications, Duhuo-Tisheng Tang and Danggui-Sini Tang (Wu et al., 2008). However, contradictory reports suggest that that digital vascular response of RP patients receiving this therapy was not changed in patients consuming the above herbal combination (Appiah et al., 1997; Hirschl et al., 2004; Wu et al., 2008). Acupuncture has also be employed in the management of RP. Indeed, a randomized controlled prospective study concluded that traditional Chinese acupuncture appears to be an effective approach in relieving symptoms, particularly attack frequency, of primary RP (Appiah et al., 1997). Others have also reported that auricular electroacupuncture could be helpful in reducing severity and frequency of RP attacks (Schlager et al., 2011). However, meta-analysis and systematic review of the literature does not conclusively support the use of acupuncture in the management of RP (Malenfant et al., 2009; Huisstede et al., 2011).

Laser therapy has also received some attention. A randomized placebo-controlled double-blind crossover study involving 48 patients shows that low level laser therapy could reduce frequency and severity of RP attacks (Hirschl et al., 2004). Findings of this study are consistent with those of another double-blind study that appeared in the same year (al-Awami et al., 2004). High-peak power laser treatment has also been reported to reduce the frequency and severity of attacks in a patient suffering from Scleroderma and RP (St Surin-Lord and Obagi, 2011).

It is important to note that surgical therapies may be considered as an option of treatment (Landry, 2013). These therapies include thoracic sympathectomy, hand stripping, and nerve stimulation (Kaada, 1982; Balogh et al., 2002; Coveliers et al., 2011). Although invasive, these are considered to be successful in pain reduction and ulcer healing (Landry, 2013). Finally, fat grafting in the patient’s hands is a new and rather unconventional surgical therapy for RP patients (Bank et al., 2014). This novel treatment originated from clinical improvements observed after fat grafting in hands suffering from burns and radiation dermatitis (Rigotti et al., 2007). When it was later “tested” on a group of RP patients, the results were encouraging and included alleviation of pain, decrease of ulcers, and decline in cold attacks (Bank et al., 2014). Although the mechanism by which fat grafting caused these effects is largely unclear, it is hypothesized that pathways involving neoangiogenesis and stem cells are likely implicated (Bank et al., 2014).

The variability of the treatments and their altered efficacies calls for urgent and concerted efforts to better understand the molecular mechanisms underlying the disease, as well as to develop more targeted and efficient drugs. These drugs may include blockers of α_2-ARs_ as well as inhibitors of PTKs and Rho kinase (Lambova and Muller-Ladner, 2009). Indeed, the first proof of concept for ameliorating RP attacks by blocking α_2_-ARs came from a study by Freedman et al. (1995). This paper showed that yohimbine, α_2_-AR antagonist, but not prazosin, α_1_-AR antagonist, can significantly attenuate vasospastic attacks of RP. More specifically, a double-blind, placebo-controlled, randomized crossover study investigated the efficacy of OPC-28326, a selective α-AR antagonist with preferential binding to the α_2C_-AR subtype, in recovery from cold-induced vasospasm in secondary RP patients. This study showed that OPC-28326 is able to improve digital blood flow after acute cold challenge in patients with RP secondary to scleroderma (Wise et al., 2004). Another phase II, randomized, double-blind, crossover, single-dose, placebo-controlled, study also tested the efficacy of ORM-12741, a potent α_2C_-AR antagonist. Interestingly, findings of this study were unexpected in that ORM-12741 prolonged, rather than shortened, the duration of the cold-induced constriction of digital arteries evident by delayed rewarming after a cold challenge (Herrick et al., 2014). The reasons for this rather unexpected result remain unclear and thus, further research is warranted to better understand the intriguing biology of α_2C_-AR especially as it related to RP pathophysiology.

## Conclusion and Perspectives

Despite the exponentially growing research and biomedical advances, a definitive and curative treatment for RP still poses a real and elusive challenge. Although many aspects and factors contributing to this disease have been dissected, the molecular mechanisms underlying the onset and progression of RP still require further investigations. This is, in no small part, due to the multifactorial etiology (hormonal, neuronal, and endothelial) of the disease. Another challenge is the absence of an appropriate animal model of the disease. The fact that α_2C_-AR is expressed in many brain regions such as the olfactory bulb and the cerebral cortex further complicates the hunt for an RP-specific drug. This is especially challenging because α_2C_-ARs are also implicated in presynaptic regulation of the heart. Thus, targeting α_2C_-ARs in an attempt to treat RP would not be most suitable, since it will affect the heart and brain as well. However, it is tempting to speculate that applying topical creams containing α_2C_-ARs blockers to affected body parts could be beneficial, and likely with fewer side effects. However, rigorous basic research and clinical trials are needed to support this suggestion. So far, precaution is often effective in reducing cold-induced vasospastic attacks of RP.

## Author Contributions

All authors contributed to the writing. AHE conceived, designed, and revised the manuscript.

## Conflict of Interest Statement

The authors declare that the research was conducted in the absence of any commercial or financial relationships that could be construed as a potential conflict of interest.

## References

[B1] Abou-Raya A. Abou-Raya S. Helmii M. (2008). Statins: potentially useful in therapy of systemic sclerosis-related Raynaud’s phenomenon and digital ulcers. *J. Rheumatol.* 35 1801–1808. 18709692

[B2] Ahles A. Engelhardt S. (2014). Polymorphic variants of adrenoceptors: pharmacology, physiology, and role in disease. *Pharmacol. Rev.* 66 598–637. 10.1124/pr.113.008219 24928328

[B3] al-Awami M. Schillinger M. Maca T. Pollanz S. Minar E. (2004). Low level laser therapy for treatment of primary and secondary Raynaud’s phenomenon. *Vasa* 33 25–29. 10.1024/0301-1526.33.1.25 15061044

[B4] Anderson J. E. Held N. Wright K. (2004). Raynaud’s phenomenon of the nipple: a treatable cause of painful breastfeeding. *Pediatrics* 113 e360–e364. 10.1542/peds.113.4.e360 15060268

[B5] Appiah R. Hiller S. Caspary L. Alexander K. Creutzig A. (1997). Treatment of primary Raynaud’s syndrome with traditional Chinese acupuncture. *J. Intern. Med.* 241 119–124. 10.1046/j.1365-2796.1997.91105000.x 9077368

[B6] Bailey S. R. Eid A. H. Mitra S. Flavahan S. Flavahan N. A. (2004). Rho kinase mediates cold-induced constriction of cutaneous arteries: role of alpha2C-adrenoceptor translocation. *Circ. Res.* 94 1367–1374. 10.1161/01.RES.0000128407.45014.58 15087420

[B7] Bailey S. R. Mitra S. Flavahan S. Flavahan N. A. (2005). Reactive oxygen species from smooth muscle mitochondria initiate cold-induced constriction of cutaneous arteries. *Am. J. Physiol. Heart Circ. Physiol.* 289 H243–H250. 10.1152/ajpheart.01305.2004 15764673

[B8] Balogh B. Mayer W. Vesely M. Mayer S. Partsch H. Piza-Katzer H. (2002). Adventitial stripping of the radial and ulnar arteries in Raynaud’s disease. *J. Hand Surg Am* 27 1073–1080. 10.1053/jhsu.2002.35887 12457360

[B9] Bank J. Fuller S. M. Henry G. I. Zachary L. S. (2014). Fat grafting to the hand in patients with Raynaud phenomenon: a novel therapeutic modality. *Plast Reconstr. Surg.* 133 1109–1118. 10.1097/PRS.0000000000000104 24445877

[B10] Belch J. J. F. Ho M. (2001). “Vasospastic disorders and vasculitis,” in *Vascular and Endovascular Surgery*, eds Beard J. D. Gaines P. A. (London: WB Saunders and Company), 217–240.

[B11] Black C. M. (1995). Systemic sclerosis ‘state of the art’ 1995. *Scand. J. Rheumatol.* 24 194–196. 10.3109/03009749509100872 7481580

[B12] Block J. A. Sequeira W. (2001). Raynaud’s phenomenon. *Lancet* 357 2042–2048. 10.1016/S0140-6736(00)05118-7 11438158

[B13] Caglayan E. Huntgeburth M. Karasch T. Weihrauch J. Hunzelmann N. Krieg T. (2006). Phosphodiesterase type 5 inhibition is a novel therapeutic option in Raynaud disease. *Arch. Intern. Med.* 166 231–233. 10.1001/archinte.166.2.231 16432094

[B14] Cardelli M. B. Kleinsmith D. M. (1989). Raynaud’s phenomenon and disease. *Med. Clin. North Am.* 73 1127–1141. 10.1016/S0025-7125(16)30623-X 2671536

[B15] Carmichael J. (1998). The role of gemcitabine in the treatment of other tumours. *Br. J. Cancer* 78(Suppl. 3), 21–25. 10.1038/bjc.1998.750 PMC2062798 9717987

[B16] Chan N. N. MacAllister R. J. Colhoun H. M. Vallance P. Hingorani A. D. (2001). Changes in endothelium-dependent vasodilatation and alpha-adrenergic responses in resistance vessels during the menstrual cycle in healthy women. *J. Clin. Endocrinol. Metab.* 86 2499–2504. 10.1210/jcem.86.6.7581 11397846

[B17] Chant A. D. (1987). Exaggerated postural vasoconstrictor reflex in Raynaud’s phenomenon. *Br. Med. J. (Clin. Res. Ed.)* 295:51 10.1136/bmj.295.6589.51-a PMC1246923 3113611

[B18] Charkoudian N. (2010). Mechanisms and modifiers of reflex induced cutaneous vasodilation and vasoconstriction in humans. *J. Appl. Physiol. (*1985) 109 1221–1228. 10.1152/japplphysiol.00298.2010 20448028 PMC2963327

[B19] Charkoudian N. Stachenfeld N. (2016). Sex hormone effects on autonomic mechanisms of thermoregulation in humans. *Auton. Neurosci.* 196 75–80. 10.1016/j.autneu.2015.11.004 26674572

[B20] Cherniack M. Clive J. Seidner A. (2000). Vibration exposure, smoking, and vascular dysfunction. *Occup. Environ. Med.* 57 341–347. 10.1136/oem.57.5.341 10769300 PMC1739951

[B21] Chitaley K. Webb R. C. (2002). Microtubule depolymerization facilitates contraction of rat aorta via activation of Rho-kinase. *Vascul. Pharmacol.* 38 157–161. 10.1016/S1537-1891(02)00163-5 12402514

[B22] Chotani M. A. Flavahan S. Mitra S. Daunt D. Flavahan N. A. (2000). Silent alpha(2C)-adrenergic receptors enable cold-induced vasoconstriction in cutaneous arteries. *Am. J. Physiol. Heart Circ. Physiol.* 278 H1075–H1083. 10749700 10.1152/ajpheart.2000.278.4.H1075

[B23] Chotani M. A. Mitra S. Su B. Y. Flavahan S. Eid A. H. Clark K. R. (2004). Regulation of alpha(2)-adrenoceptors in human vascular smooth muscle cells. *Am. J. Physiol. Heart Circ. Physiol.* 286 H59–H67. 10.1152/ajpheart.00268.2003 12946937

[B24] Clifford P. C. Martin M. F. Sheddon E. J. Kirby J. D. Baird R. N. Dieppe P. A. (1980). Treatment of vasospastic disease with prostaglandin E1. *Br. Med. J.* 281 1031–1034. 10.1136/bmj.281.6247.1031 7427564 PMC1714438

[B25] Clowse M. E. Wigley F. M. (2003). Digital necrosis related to carboplatin and gemcitabine therapy in systemic sclerosis. *J. Rheumatol.* 30 1341–1343. 12784412

[B26] Coleiro B. Marshall S. E. Denton C. P. Howell K. Blann A. Welsh K. I. (2001). Treatment of Raynaud’s phenomenon with the selective serotonin reuptake inhibitor fluoxetine. *Rheumatology (Oxford)* 40 1038–1043. 10.1093/rheumatology/40.9.1038 11561116

[B27] Coveliers H. M. Hoexum F. Nederhoed J. H. Wisselink W. Rauwerda J. A. (2011). Thoracic sympathectomy for digital ischemia: a summary of evidence. *J. Vasc. Surg.* 54 273–277. 10.1016/j.jvs.2011.01.069 21652164

[B28] Coward J. Maisey N. Cunningham D. (2005). The effects of capecitabine in Raynaud’s disease: a case report. *Ann. Oncol.* 16 835–836. 10.1093/annonc/mdi144 15788442

[B29] Csiki Z. Garai I. Shemirani A. H. Papp G. Zsori K. S. Andras C. (2011). The effect of metoprolol alone and combined metoprolol-felodipin on the digital microcirculation of patients with primary Raynaud’s syndrome. *Microvasc. Res.* 82 84–87. 10.1016/j.mvr.2011.04.004 21515290

[B30] D’Alessandro V. Errico M. Varriale A. Greco A. De Cata A. Carnevale V. (2003). [Case report: Acro-necrosis of the upper limbs caused by gemcitabine therapy]. *Clin. Ter.* 154 207–210. 12910811

[B31] Dziadzio M. Denton C. P. Smith R. Howell K. Blann A. Bowers E. (1999). Losartan therapy for Raynaud’s phenomenon and scleroderma: clinical and biochemical findings in a fifteen-week, randomized, parallel-group, controlled trial. *Arthritis Rheum.* 42 2646–2655. 10.1002/1529-0131(199912)42:12<2646::AID-ANR21>3.0.CO;2-T 10616013

[B32] Easter M. J. Marshall J. M. (2005). Contribution of prostanoids to endothelium-dependent vasodilatation in the digital circulation of women with primary Raynaud’s disease. *Clin. Sci. (Lond).* 109 45–54. 10.1042/CS20040262 15769250

[B33] Eid A. H. Chotani M. A. Mitra S. Miller T. J. Flavahan N. A. (2008). Cyclic AMP acts through Rap1 and JNK signaling to increase expression of cutaneous smooth muscle alpha2C-adrenoceptors. *Am. J. Physiol. Heart Circ. Physiol.* 295 H266–H272. 10.1152/ajpheart.00084.2008 18487435 PMC2494767

[B34] Eid A. H. Maiti K. Mitra S. Chotani M. A. Flavahan S. Bailey S. R. (2007). Estrogen increases smooth muscle expression of alpha2C-adrenoceptors and cold-induced constriction of cutaneous arteries. *Am. J. Physiol. Heart Circ. Physiol.* 293 H1955–H1961. 10.1152/ajpheart.00306.2007 17644575

[B35] English K. M. Jones R. D. Jones T. H. Morice A. H. Channer K. S. (2001). Gender differences in the vasomotor effects of different steroid hormones in rat pulmonary and coronary arteries. *Horm. Metab. Res.* 33 645–652. 10.1055/s-2001-18689 11733866

[B36] Ennis H. Hughes M. Anderson M. E. Wilkinson J. Herrick A. L. (2016). Calcium channel blockers for primary Raynaud’s phenomenon. *Cochrane Database Syst. Rev.* 2: CD002069 10.1002/14651858.CD002069.pub5 PMC7065590 26914257

[B37] Falappa P. Magnavita N. Bergamaschi A. Colavita N. (1982). Angiographic study of digital arteries in workers exposed to vinyl chloride. *Br. J. Ind. Med.* 39 169–172. 7066233 10.1136/oem.39.2.169 PMC1008965

[B38] Flavahan N. A. (2015). A vascular mechanistic approach to understanding Raynaud phenomenon. *Nat. Rev. Rheumatol.* 11 146–158. 10.1038/nrrheum.2014.195 25536485

[B39] Fletcher D. A. Mullins R. D. (2010). Cell mechanics and the cytoskeleton. *Nature* 463 485–492. 10.1038/nature08908 20110992 PMC2851742

[B40] Fontana L. Marion M. J. Ughetto S. Catilina P. (2006). Glutathione S-transferase M1 and GST T1 genetic polymorphisms and Raynaud’s phenomenon in French vinyl chloride monomer-exposed workers. *J. Hum. Genet.* 51 879–886. 10.1007/s10038-006-0038-9 16977343

[B41] Fraenkel L. Zhang Y. Chaisson C. E. Evans S. R. Wilson P. W. Felson D. T. (1998). The association of estrogen replacement therapy and the Raynaud phenomenon in postmenopausal women. *Ann. Intern. Med.* 129 208–211. 10.7326/0003-4819-129-3-199808010-00009 9696729

[B42] Freedman R. R. Baer R. P. Mayes M. D. (1995). Blockade of vasospastic attacks by alpha 2-adrenergic but not alpha 1-adrenergic antagonists in idiopathic Raynaud’s disease. *Circulation* 92 1448–1451. 10.1161/01.CIR.92.6.1448 7664425

[B43] Freedman R. R. Moten M. Migaly P. Mayes M. (1993). Cold-induced potentiation of alpha 2-adrenergic vasoconstriction in primary Raynaud’s disease. *Arthritis Rheum.* 36 685–690. 10.1002/art.1780360517 8387786

[B44] Freedman R. R. Sabharwal S. C. Moten M. Migaly P. (1992). Local temperature modulates alpha 1- and alpha 2-adrenergic vasoconstriction in men. *Am. J. Physiol.* 263 H1197–H1200. 1329562 10.1152/ajpheart.1992.263.4.H1197

[B45] Funauchi M. Kishimoto K. Shimazu H. Nagare Y. Hino S. Yano T. (2009). Effects of bosentan on the skin lesions: an observational study from a single center in Japan. *Rheumatol. Int.* 29 769–775. 10.1007/s00296-008-0789-z 19037604

[B46] Furspan P. B. Chatterjee S. Freedman R. R. (2004). Increased tyrosine phosphorylation mediates the cooling-induced contraction and increased vascular reactivity of Raynaud’s disease. *Arthritis Rheum.* 50 1578–1585. 10.1002/art.20214 15146428

[B47] Furspan P. B. Chatterjee S. Mayes M. D. Freedman R. R. (2005). Cooling-induced contraction and protein tyrosine kinase activity of isolated arterioles in secondary Raynaud’s phenomenon. *Rheumatology* 44 488–494. 10.1093/rheumatology/keh517 15695304

[B48] Garner R. Kumari R. Lanyon P. Doherty M. Zhang W. (2015). Prevalence, risk factors and associations of primary Raynaud’s phenomenon: systematic review and meta-analysis of observational studies. *BMJ Open* 5: e006389 10.1136/bmjopen-2014-006389 PMC4368987 25776043

[B49] Gentric A. Blaschek M. A. Le Noach J. F. Johanet C. Jouquan J. Lamour A. (1990). Serological arguments for classifying Raynaud’s phenomenon as idiopathic. *J. Rheumatol.* 17 1177–1181. 2290158

[B50] Gerbracht D. D. Steen V. D. Ziegler G. L. Medsger T. A. Jr. Rodnan G. P. (1985). Evolution of primary Raynaud’s phenomenon (Raynaud’s disease) to connective tissue disease. *Arthritis Rheum.* 28 87–92. 10.1002/art.1780280114 3871330

[B51] Glendenning J. L. Barbachano Y. Norman A. R. Dearnaley D. P. Horwich A. Huddart R. A. (2010). Long-term neurologic and peripheral vascular toxicity after chemotherapy treatment of testicular cancer. *Cancer* 116 2322–2331. 10.1002/cncr.24981 20225230

[B52] Gliddon A. E. Dore C. J. Black C. M. McHugh N. Moots R. Denton C. P. (2007). Prevention of vascular damage in scleroderma and autoimmune Raynaud’s phenomenon: a multicenter, randomized, double-blind, placebo-controlled trial of the angiotensin-converting enzyme inhibitor quinapril. *Arthritis Rheum.* 56 3837–3846. 10.1002/art.22965 17968938

[B53] Gottschling S. Meyer S. Reinhard H. Krenn T. Graf N. (2004). First report of a vincristine dose-related Raynaud’s phenomenon in an adolescent with malignant brain tumor. *J. Pediatr. Hematol. Oncol.* 26 768–769. 10.1097/00043426-200411000-00017 15543016

[B54] Greenstein D. Jeffcote N. Ilsley D. Kester R. C. (1996). The menstrual cycle and Raynaud’s phenomenon. *Angiology* 47 427–436. 10.1177/000331979604700501 8644939

[B55] Guimaraes S. Moura D. (2001). Vascular adrenoceptors: an update. *Pharmacol. Rev.* 53 319–356. 11356987

[B56] Halawa B. (2001). [Calcium channel blockers in the treatment of cardiovascular disease]. *Pol. Merkur. Lekarski* 11 83–87. 11579840

[B57] Hall A. (1998). Rho GTPases and the actin cytoskeleton. *Science* 279 509–514. 10.1126/science.279.5350.509 9438836

[B58] Hall A. H. (2002). Chronic arsenic poisoning. *Toxicol. Lett.* 128 69–72. 10.1016/S0378-4274(01)00534-3 11869818

[B59] Hansen S. W. Olsen N. (1989). Raynaud’s phenomenon in patients treated with cisplatin, vinblastine, and bleomycin for germ cell cancer: measurement of vasoconstrictor response to cold. *J. Clin. Oncol.* 7 940–942. 2472472 10.1200/JCO.1989.7.7.940

[B60] Heidrich H. (2010). Functional vascular diseases: Raynaud’s syndrome, acrocyanosis and erythromelalgia. *Vasa* 39 33–41. 10.1024/0301-1526/a000003 20186674

[B61] Henness S. Wigley F. M. (2007). Current drug therapy for scleroderma and secondary Raynaud’s phenomenon: evidence-based review. *Curr. Opin. Rheumatol.* 19 611–618. 10.1097/BOR.0b013e3282f13137 17917543

[B62] Herrick A. L. (2012). The pathogenesis, diagnosis and treatment of Raynaud phenomenon. *Nat. Rev. Rheumatol.* 8 469–479. 10.1038/nrrheum.2012.96 22782008

[B63] Herrick A. L. Murray A. K. Ruck A. Rouru J. Moore T. L. Whiteside J. (2014). A double-blind, randomized, placebo-controlled crossover trial of the alpha2C-adrenoceptor antagonist ORM-12741 for prevention of cold-induced vasospasm in patients with systemic sclerosis. *Rheumatology (Oxford)* 53 948–952. 10.1093/rheumatology/ket421 24489014

[B64] Hirschl M. Katzenschlager R. Francesconi C. Kundi M. (2004). Low level laser therapy in primary Raynaud’s phenomenon–results of a placebo controlled, double blind intervention study. *J. Rheumatol.* 31 2408–2412. 15570642

[B65] Holstein A. Batge R. Egberts E. H. (2010). Gemcitabine induced digital ischaemia and necrosis. *Eur. J. Cancer Care (Engl).* 19 408–409. 10.1111/j.1365-2354.2008.01057.x 19490003

[B66] Huisstede B. M. Hoogvliet P. Paulis W. D. van Middelkoop M. Hausman M. Coert J. H. (2011). Effectiveness of interventions for secondary Raynaud’s phenomenon: a systematic review. *Arch. Phys. Med. Rehabil.* 92 1166–1180. 10.1016/j.apmr.2011.01.022 21704799

[B67] Hurt C. M. Feng F. Y. Kobilka B. (2000). Cell-type specific targeting of the alpha 2c-adrenoceptor. Evidence for the organization of receptor microdomains during neuronal differentiation of PC12 cells. *J. Biol. Chem.* 275 35424–35431. 10.1074/jbc.M006241200 10906149

[B68] Jackson C. M. (2006). The patient with cold hands: understanding Raynaud’s disease. *JAAPA* 19 34–38. 10.1097/01720610-200611000-00006 17124789

[B69] Janini S. D. Scott D. G. Coppock J. S. Bacon P. A. Kendall M. J. (1988). Enalapril in Raynaud’s phenomenon. *J. Clin. Pharm. Ther.* 13 145–150. 10.1111/j.1365-2710.1988.tb00171.x 2839529

[B70] Jeyaraj S. C. Chotani M. A. Mitra S. Gregg H. E. Flavahan N. A. Morrison K. J. (2001). Cooling evokes redistribution of alpha2C-adrenoceptors from Golgi to plasma membrane in transfected human embryonic kidney 293 cells. *Mol. Pharmacol.* 60 1195–1200. 11723226 10.1124/mol.60.6.1195

[B71] Jeyaraj S. C. Unger N. T. Eid A. H. Mitra S. Paul El-Dahdah N. Quilliam L. A. (2012). Cyclic AMP-Rap1A signaling activates RhoA to induce alpha(2c)-adrenoceptor translocation to the cell surface of microvascular smooth muscle cells. *Am. J. Physiol. Cell Physiol.* 303 C499–C511. 10.1152/ajpcell.00461.2011 22621783 PMC3468345

[B72] Johnson J. M. Kellogg D. L. Jr. (2010). Local thermal control of the human cutaneous circulation. *J. Appl. Physiol. (*1985) 109 1229–1238. 10.1152/japplphysiol.00407.2010 20522732 PMC2963328

[B73] Kaada B. (1982). Vasodilation induced by transcutaneous nerve stimulation in peripheral ischemia (Raynaud’s phenomenon and diabetic polyneuropathy). *Eur. Heart J.* 3 303–314. 6982164 10.1093/oxfordjournals.eurheartj.a061312

[B74] Kawald A. Burmester G. R. Huscher D. Sunderkotter C. Riemekasten G. (2008). Low versus high-dose iloprost therapy over 21 days in patients with secondary Raynaud’s phenomenon and systemic sclerosis: a randomized, open, single-center study. *J. Rheumatol.* 35 1830–1837. 18634152

[B75] Koltringer P. Langsteger W. Pierer G. Lind P. Klima G. Reisecker F. (1991). [Effect of metoprolol on microcirculation and blood viscoelasticity]. *Acta Med. Austriaca* 18 75–77. 1950383

[B76] Kristensen J. K. (1979). Local regulation of digital blood flow in generalized scleroderma. *J. Invest. Dermatol.* 72 235–240. 10.1111/1523-1747.ep12531698 458185

[B77] Kuhar C. G. Mesti T. Zakotnik B. (2010). Digital ischemic events related to gemcitabine: report of two cases and a systematic review. *Radiol. Oncol.* 44 257–261. 10.2478/v10019-010-0020-1 22933925 PMC3423709

[B78] Kundu D. Abraham D. Black C. M. Denton C. P. Bruckdorfer K. R. (2014). Reduced levels of S-nitrosothiols in plasma of patients with systemic sclerosis and Raynaud’s phenomenon. *Vascul. Pharmacol.* 63 178–181. 10.1016/j.vph.2014.09.003 25446164 PMC4265732

[B79] Lagerkvist B. Linderholm H. Nordberg G. F. (1986). Vasospastic tendency and Raynaud’s phenomenon in smelter workers exposed to arsenic. *Environ. Res.* 39 465–474. 10.1016/S0013-9351(86)80070-6 3956470

[B80] Lambova S. N. Muller-Ladner U. (2009). New lines in therapy of Raynaud’s phenomenon. *Rheumatol. Int.* 29 355–363. 10.1007/s00296-008-0792-4 19037602

[B81] Landry G. J. (2013). Current medical and surgical management of Raynaud’s syndrome. *J. Vasc. Surg.* 57 1710–1716. 10.1016/j.jvs.2013.03.012 23618525

[B82] Le Quentrec P. Lefebvre M. L. (1991). Double-blind placebo-controlled trial of buflomedil in the treatment of Raynaud’s phenomenon: six-month follow-up. *Angiology* 42 289–295. 10.1177/000331979104200405 1673051

[B83] Lee E. Y. Park J. K. Lee W. Kim Y. K. Park C. S. Giles J. T. (2014). Head-to-head comparison of udenafil vs amlodipine in the treatment of secondary Raynaud’s phenomenon: a double-blind, randomized, cross-over study. *Rheumatology (Oxford)* 53 658–664. 10.1093/rheumatology/ket417 24352340

[B84] Leppert J. Aberg H. Levin K. Ringqvist I. (1990). Lower serum magnesium level after exposure to cold in women with primary Raynaud’s phenomenon. *J. Intern. Med.* 228 235–239. 10.1111/j.1365-2796.1990.tb00224.x 2401874

[B85] Li T. Xiao X. Zhang J. Zhu Y. Hu Y. Zang J. (2014). Age and sex differences in vascular responsiveness in healthy and trauma patients: contribution of estrogen receptor-mediated Rho kinase and PKC pathways. *Am. J. Physiol. Heart Circ. Physiol.* 306 H1105–H1115. 10.1152/ajpheart.00645.2013 24531808

[B86] Linnemann B. Erbe M. (2016). Raynaud’s phenomenon and digital ischaemia–pharmacologic approach and alternative treatment options. *Vasa* 45 201–212. 10.1024/0301-1526/a000526 27129065

[B87] MacDonald E. Kobilka B. K. Scheinin M. (1997). Gene targeting–homing in on alpha 2-adrenoceptor-subtype function. *Trends Pharmacol. Sci.* 18 211–219. 10.1016/S0165-6147(97)01063-8 9227000

[B88] Malenfant D. Catton M. Pope J. E. (2009). The efficacy of complementary and alternative medicine in the treatment of Raynaud’s phenomenon: a literature review and meta-analysis. *Rheumatology (Oxford)* 48 791–795. 10.1093/rheumatology/kep039 19433434

[B89] Maricq H. R. Carpentier P. H. Weinrich M. C. Keil J. E. Franco A. Drouet P. (1993). Geographic variation in the prevalence of Raynaud’s phenomenon: Charleston, SC, USA, vs Tarentaise, Savoie, France. *J. Rheumatol.* 20 70–76. 8441170

[B90] Maricq H. R. Darke C. S. Archibald R. M. Leroy E. C. (1978). In vivo observations of skin capillaries in workers exposed to vinyl chloride. An English-American comparison. *Br. J. Ind. Med.* 35 1–7. 629883 10.1136/oem.35.1.1 PMC1008316

[B91] Maricq H. R. Johnson M. N. Whetstone C. L. LeRoy E. C. (1976). Capillary abnormalities in polyvinyl chloride production workers. Examination by in vivo microscopy. *JAMA* 236 1368–1371. 10.1001/jama.236.12.1368 989092

[B92] Marshall A. J. Roberts C. J. Barritt D. W. (1976). Raynaud’s phenomenon as side effect of beta-blockers in hypertension. *Br. Med. J.* 1 1498–1499. 10.1136/bmj.1.6024.1498 6109 PMC1640736

[B93] Maverakis E. Patel F. Kronenberg D. G. Chung L. Fiorentino D. Allanore Y. (2014). International consensus criteria for the diagnosis of Raynaud’s phenomenon. *J. Autoimmun.* 4 60–65. 10.1016/j.jaut.2014.01.020 PMC4018202 24491823

[B94] Mayes M. D. (1999). Epidemiologic studies of environmental agents and systemic autoimmune diseases. *Environ. Health Perspect.* 107(Suppl. 5), 743–748. 10.2307/3434336 10502540 PMC1566245

[B95] McCance K. L. Huether S. E. (2013). *Pathophysiology: The Biologic Basis for Disease in Adults and Children*. Amsterdam: Elsevier.

[B96] McNeill A. M. Leslie F. M. Krause D. N. Duckles S. P. (1999). Gender difference in levels of alpha2-adrenoceptor mRNA in the rat tail artery. *Eur. J. Pharmacol.* 366 233–236. 10.1016/S0014-2999(98)00948-0 10082204

[B97] Milio G. Corrado E. Genova C. Amato C. Raimondi F. Almasio P. L. (2006). Iloprost treatment in patients with Raynaud’s phenomenon secondary to systemic sclerosis and the quality of life: a new therapeutic protocol. *Rheumatology (Oxford)* 45 999–1004. 10.1093/rheumatology/kel038 16484290

[B98] Mohokum M. Hartmann P. Schlattmann P. (2012). The association of Raynaud syndrome with beta-blockers: a meta-analysis. *Angiology* 63 535–540. 10.1177/0003319711432861 22261435

[B99] Mohrland J. S. Porter J. M. Smith E. A. Belch J. Simms M. H. (1985). A multiclinic, placebo-controlled, double-blind study of prostaglandin E1 in Raynaud’s syndrome. *Ann. Rheum. Dis.* 44 754–760. 10.1136/ard.44.11.754 3904643 PMC1001768

[B100] Motawea H. K. Jeyaraj S. C. Eid A. H. Mitra S. Unger N. T. Ahmed A. A. (2013). Cyclic AMP-Rap1A signaling mediates cell surface translocation of microvascular smooth muscle alpha2C-adrenoceptors through the actin-binding protein filamin-2. *Am. J. Physiol. Cell Physiol.* 305 C829–C845. 10.1152/ajpcell.00221.2012 23864608 PMC3798683

[B101] Muir A. H. Robb R. McLaren M. Daly F. Belch J. J. (2002). The use of Ginkgo biloba in Raynaud’s disease: a double-blind placebo-controlled trial. *Vasc. Med.* 7 265–267. 10.1191/1358863x02vm455oa 12710841

[B102] Neumeister M. W. Webb K. N. Romanelli M. (2014). Minimally invasive treatment of Raynaud phenomenon: the role of botulinum type A. *Hand Clin.* 30 17–24. 10.1016/j.hcl.2013.09.006 24286738

[B103] Nordberg G. F. Fowler B. A. Nordberg M. Friberg L. (2014). *Handbook on the Toxicology of Metals.* Amsterdam: Academic Press.

[B104] Nyronen T. Pihlavisto M. Peltonen J. M. Hoffren A. M. Varis M. Salminen T. (2001). Molecular mechanism for agonist-promoted alpha(2A)-adrenoceptor activation by norepinephrine and epinephrine. *Mol. Pharmacol.* 59 1343–1354. 11306720 10.1124/mol.59.5.1343

[B105] Olsen N. Petring O. U. Rossing N. (1987). Exaggerated postural vasoconstrictor reflex in Raynaud’s phenomenon. *Br. Med. J. (Clin. Res. Ed.)* 294 1186–1188. 10.1136/bmj.294.6581.1186 PMC1246353 3109573

[B106] Pawlowski M. Saraswathi S. Motawea H. K. Chotani M. A. Kloczkowski A. (2014). In silico modeling of human alpha2C-adrenoreceptor interaction with filamin-2. *PLoS ONE* 9:e103099 10.1371/journal.pone.0103099 PMC4128582 25110951

[B107] Pistorius M. A. Planchon B. Schott J. J. Lemarec H. (2006). [Heredity and genetic aspects of Raynaud’s disease]. *J. Mal. Vasc.* 31 10–15. 10.1016/S0398-0499(06)76512-X 16609626

[B108] Polonia J. J. Paiva M. Q. Guimaraes S. (1985). Pharmacological characterization of postsynaptic alpha-adrenoceptor subtypes in five different dog arteries in-vitro. *J. Pharm. Pharmacol.* 37 205–208. 10.1111/j.2042-7158.1985.tb05043.x 2858570

[B109] Pope J. Fenlon D. Thompson A. Shea B. Furst D. Wells G. (2000). Iloprost and cisaprost for Raynaud’s phenomenon in progressive systemic sclerosis. *Cochrane Database Syst. Rev.* 2:CD000953 10.1002/14651858.CD000953 PMC7032888 10796395

[B110] Poredos P. Poredos P. (2016). Raynaud’s syndrome: a neglected disease. *Int. Angiol.* 35 117–121. 25673314

[B111] Prete M. Fatone M. C. Favoino E. Perosa F. (2014). Raynaud’s phenomenon: from molecular pathogenesis to therapy. *Autoimmun. Rev.* 13 655–667. 10.1016/j.autrev.2013.12.001 24418302

[B112] Rademaker M. Thomas R. H. Provost G. Beacham J. A. Cooke E. D. Kirby J. D. (1987). Prolonged increase in digital blood flow following iloprost infusion in patients with systemic sclerosis. *Postgrad. Med. J.* 63 617–620. 10.1136/pgmj.63.742.617 2447572 PMC2428395

[B113] Reslan O. M. Khalil R. A. (2012). Vascular effects of estrogenic menopausal hormone therapy. *Rev. Recent Clin. Trials* 7 47–70. 10.2174/157488712799363253 21864249 PMC3227781

[B114] Rigotti G. Marchi A. Galie M. Baroni G. Benati D. Krampera M. (2007). Clinical treatment of radiotherapy tissue damage by lipoaspirate transplant: a healing process mediated by adipose-derived adult stem cells. *Plast Reconstr. Surg.* 119 1409–1422;discussion1423–1404. 10.1097/01.prs.0000256047.47909.71 17415234

[B115] Roustit M. Khouri C. Blaise S. Villier C. Carpentier P. Cracowski J. L. (2014). [Pharmacology of Raynaud’s phenomenon]. *Therapie* 69 115–128. 10.2515/therapie/2013068 24926630

[B116] Rychlik-Golema W. Mastej K. Adamiec R. (2006). The role of endothelin-1 and selected cytokines in the pathogenesis of Raynaud’s phenomenon associated with systemic connective tissue diseases. *Int. Angiol.* 25 221–227. 16763543

[B117] Saif M. W. Agarwal A. Hellinger J. Park D. J. Volkmann E. (2016). Scleroderma in a patient on capecitabine: is this a variant of hand-foot syndrome? *Cureus* 8:e663 10.7759/cureus.663 PMC4968779 27493845

[B118] Sallinen J. Link R. E. Haapalinna A. Viitamaa T. Kulatunga M. Sjoholm B. (1997). Genetic alteration of alpha 2C-adrenoceptor expression in mice: influence on locomotor, hypothermic, and neurochemical effects of dexmedetomidine, a subtype-nonselective alpha 2-adrenoceptor agonist. *Mol. Pharmacol.* 51 36–46. 9016344 10.1124/mol.51.1.36

[B119] Schlager O. Gschwandtner M. E. Mlekusch I. Herberg K. Frohner T. Schillinger M. (2011). Auricular electroacupuncture reduces frequency and severity of Raynaud attacks. *Wien. Klin. Wochenschr.* 123 112–116. 10.1007/S00508-011-1531-5 21327676

[B120] Scorza R. Caronni M. Mascagni B. Berruti V. Bazzi S. Micallef E. (2001). Effects of long-term cyclic iloprost therapy in systemic sclerosis with Raynaud’s phenomenon. A randomized, controlled study. *Clin. Exp. Rheumatol.* 19 503–508. 11579708

[B121] Selenko-Gebauer N. Duschek N. Minimair G. Stingl G. Karlhofer F. (2006). Successful treatment of patients with severe secondary Raynaud’s phenomenon with the endothelin receptor antagonist bosentan. *Rheumatology (Oxford)* 45(Suppl. 3), iii45–iii48. 10.1093/rheumatology/kel290 16987835

[B122] Sharathkumar A. A. Castillo-Caro P. (2011). Primary Raynaud’s phenomenon in an infant: a case report and review of literature. *Pediatr. Rheumatol. Online J.* 9:16 10.1186/1546-0096-9-16 PMC3162536 21767369

[B123] Smith L. Polsky D. Franks A. G. Jr. (2012). Botulinum toxin-A for the treatment of Raynaud syndrome. *Arch. Dermatol.* 148 426–428. 10.1001/archdermatol.2011.1144 22508867

[B124] Spengler M. I. Svetaz M. J. Leroux M. B. Leiva M. L. Bottai H. M. (2004). Association between capillaroscopy, haemorheological variables and plasma proteins in patients bearing Raynaud’s phenomenon. *Clin. Hemorheol. Microcirc.* 30 17–24. 14967879

[B125] St Surin-Lord S. Obagi S. (2011). Scleroderma and raynaud’s phenomenon improve with high-peak power laser therapy: a case report. *Dermatol. Surg.* 37 1531–1535. 10.1111/j.1524-4725.2011.02093.x 21790846

[B126] Stewart M. Morling J. R. (2012). Oral vasodilators for primary Raynaud’s phenomenon. *Cochrane Database Syst. Rev.* 7:CD006687 10.1002/14651858.CD006687.pub3 PMC6718219 22786498

[B127] Susol E. MacGregor A. J. Barrett J. H. Wilson H. Black C. Welsh K. (2000). A two-stage, genome-wide screen for susceptibility loci in primary Raynaud’s phenomenon. *Arthritis Rheum.* 43 1641–1646. 10.1002/1529-0131(200007)43:7<1641::AID-ANR30>3.0.CO;2-Y 10902770

[B128] Tan F. K. Arnett F. C. (2000). Genetic factors in the etiology of systemic sclerosis and Raynaud phenomenon. *Curr. Opin. Rheumatol.* 12 511–519. 10.1097/00002281-200011000-00007 11092201

[B129] Thompson A. E. Pope J. E. (2005). Calcium channel blockers for primary Raynaud’s phenomenon: a meta-analysis. *Rheumatology (Oxford)* 44 145–150. 10.1093/rheumatology/keh390 15546967

[B130] Tosi S. Marchesoni A. Messina K. Bellintani C. Sironi G. Faravelli C. (1987). Treatment of Raynaud’s phenomenon with captopril. *Drugs Exp. Clin. Res.* 13 37–42. 3297593

[B131] Vanhoutte P. M. (1980). “Physical factors and regulation of vascular smooth muscle function,” in *Handbook of Physiology*, eds Bohr D. F. Somlyo A. P. Sparks H. V. (Washington, DC: The American Physiological Society), 443–474.

[B132] Venat-Bouvet L. Ly K. Szelag J. C. Martin J. Labourey J. L. Genet D. (2003). Thrombotic microangiopathy and digital necrosis: two unrecognized toxicities of gemcitabine. *Anticancer. Drugs* 14 829–832. 10.1097/01.cad.0000098998.92896.01 14597878

[B133] Vogelzang N. J. Bosl G. J. Johnson K. Kennedy B. J. (1981). Raynaud’s phenomenon: a common toxicity after combination chemotherapy for testicular cancer. *Ann. Intern. Med.* 95 288–292. 10.7326/0003-4819-95-3-288 6168223

[B134] von Zastrow M. Kobilka B. K. (1994). Antagonist-dependent and -independent steps in the mechanism of adrenergic receptor internalization. *J. Biol. Chem.* 269 18448–18452. 7518433

[B135] White C. R. Haidekker M. A. Stevens H. Y. Frangos J. A. (2004). Extracellular signal-regulated kinase activation and endothelin-1 production in human endothelial cells exposed to vibration. *J. Physiol.* 555 565–572. 10.1113/jphysiol.2003.059899 14724194 PMC1664844

[B136] Wigley F. M. (2002). Clinical practice. Raynaud’s phenomenon. *N. Engl. J. Med.* 347 1001–1008. 10.1056/NEJMcp013013 12324557

[B137] Wigley F. M. Flavahan N. A. (2016). Raynaud’s phenomenon. *N. Engl. J. Med.* 375 556–565. 10.1056/NEJMra1507638 27509103

[B138] Wigley F. M. Wise R. A. Seibold J. R. McCloskey D. A. Kujala G. Medsger T. A. (1994). Intravenous iloprost infusion in patients with Raynaud phenomenon secondary to systemic sclerosis. A multicenter, placebo-controlled, double-blind study. *Ann. Intern. Med.* 120 199–206. 10.7326/0003-4819-120-3-199402010-00004 7506013

[B139] William N. R. Markowitz S. B. (2007). *Environmental and Occupational Medicine*. Philadelphia: Lippincott Williams & Wilkins.

[B140] Wilson R. H. McCormick W. E. Tatum C. F. Creech J. L. (1967). Occupational acroosteolysis. Report of 31 cases. *JAMA* 201 577–581. 10.1001/jama.1967.03130080019005 5006758

[B141] Wise R. A. Wigley F. M. White B. Leatherman G. Zhong J. Krasa H. (2004). Efficacy and tolerability of a selective alpha(2C)-adrenergic receptor blocker in recovery from cold-induced vasospasm in scleroderma patients: a single-center, double-blind, placebo-controlled, randomized crossover study. *Arthritis Rheum.* 50 3994–4001. 10.1002/art.20665 15593189

[B142] Wood H. M. Ernst M. E. (2006). Renin-angiotensin system mediators and Raynaud’s phenomenon. *Ann. Pharmacother.* 40 1998–2002. 10.1345/aph.1H201 17003081

[B143] Wu Y. J. Luo S. F. Yang S. H. Chen J. Y. Yu K. H. See L. C. (2008). Vascular response of Raynaud’s phenomenon to nifedipine or herbal medication (duhuo-tisheng tang with danggui-sini tang): a preliminary study. *Chang Gung Med. J.* 31 492–502. 19097597

[B144] Yamada Y. Suzuki K. Nobata H. Kawai H. Wakamatsu R. Miura N. (2014). Gemcitabine-induced hemolytic uremic syndrome mimicking scleroderma renal crisis presenting with Raynaud’s phenomenon, positive antinuclear antibodies and hypertensive emergency. *Intern. Med.* 53 445–448. 10.2169/internalmedicine.53.1160 24583433

[B145] Zamora M. R. O’Brien R. F. Rutherford R. B. Weil J. V. (1990). Serum endothelin-1 concentrations and cold provocation in primary Raynaud’s phenomenon. *Lancet* 336 1144–1147. 10.1016/0140-6736(90)92766-B 1978025

[B146] Zhang X. Hu Y. Nie Z. Song Y. Pan Y. Liu Y. (2015). Treatment of Raynaud’s phenomenon with botulinum toxin type A. *Neurol. Sci.* 36 1225–1231. 10.1007/s10072-015-2084-6 25616446

[B147] Zhao H. Lian Y. (2015). Clinical and image improvement of Raynaud’s phenomenon after botulinum toxin type A treatment. *Australas. J. Dermatol.* 56 202–205. 10.1111/ajd.12326 25817568

